# A Novel Ebola Virus VP40 Matrix Protein-Based Screening for Identification of Novel Candidate Medical Countermeasures

**DOI:** 10.3390/v13010052

**Published:** 2020-12-31

**Authors:** Ryan P. Bennett, Courtney L. Finch, Elena N. Postnikova, Ryan A. Stewart, Yingyun Cai, Shuiqing Yu, Janie Liang, Julie Dyall, Jason D. Salter, Harold C. Smith, Jens H. Kuhn

**Affiliations:** 1 OyaGen, Inc., 77 Ridgeland Road, Rochester, NY 14623, USA; rbennett@oyageninc.com (R.P.B.); rstewart6348@gmail.com (R.A.S.); jsalter@oyageninc.com (J.D.S.); 2 NIH/NIAID/DCR/Integrated Research Facility at Fort Detrick (IRF-Frederick), Frederick, MD 21702, USA; courtney.finch@nih.gov (C.L.F.); elena.postnikova2@nih.gov (E.N.P.); caiy@niaid.nih.gov (Y.C.); shuiqing.yu@nih.gov (S.Y.); janie.liang@nih.gov (J.L.); dyallj@niaid.nih.gov (J.D.)

**Keywords:** broad spectrum, Ebola virus, *Filoviridae*, filovirus, Marburg virus, MCM, VP40, sangivamycin

## Abstract

Filoviruses, such as Ebola virus and Marburg virus, are of significant human health concern. From 2013 to 2016, Ebola virus caused 11,323 fatalities in Western Africa. Since 2018, two Ebola virus disease outbreaks in the Democratic Republic of the Congo resulted in 2354 fatalities. Although there is progress in medical countermeasure (MCM) development (in particular, vaccines and antibody-based therapeutics), the need for efficacious small-molecule therapeutics remains unmet. Here we describe a novel high-throughput screening assay to identify inhibitors of Ebola virus VP40 matrix protein association with viral particle assembly sites on the interior of the host cell plasma membrane. Using this assay, we screened nearly 3000 small molecules and identified several molecules with the desired inhibitory properties. In secondary assays, one identified compound, sangivamycin, inhibited not only Ebola viral infectivity but also that of other viruses. This finding indicates that it is possible for this new VP40-based screening method to identify highly potent MCMs against Ebola virus and its relatives.

## 1. Introduction

Filoviruses (*Mononegavirales*: *Filoviridae*) have linear non-segmented negative-sense RNA genomes (up to 19.1 kb), consisting of the canonical genes 3′-*NP-VP35-VP40-GP-VP30-VP24-L*-5′ that encode nucleoprotein (NP), polymerase cofactor (VP35), matrix protein (VP40), spike glycoprotein (GP_1,2_), transcriptional activator (VP30), RNA complex-associated protein (VP24), and large protein (L, including an RNA-directed RNA polymerase [RdRp] activity), respectively [1]. The family *Filoviridae* currently includes six genera, of which two, *Ebolavirus* and *Marburgvirus*, harbor viruses known to cause human disease [2]. Based on frequency and size of documented human filovirus disease outbreaks, two ebolaviruses (Ebola virus [EBOV] and Sudan virus [SUDV]) and one marburgvirus (Marburg virus [MARV]) are of the greatest public health concern [1]. EBOV, the etiologic agent of Ebola virus disease (EVD) [3], caused the two largest filovirus disease outbreaks on record: 28,652 cases with 11,323 deaths were reported during the 2013–2016 EVD outbreak in Western Africa; and 3481 cases with 2299 deaths occurred during a 2018–2020 EVD outbreak in the Democratic Republic of the Congo [4,5].

Relatively little progress has been made toward establishing anti-filovirus medical countermeasures (MCMs) until recently. The 2013–2016 EVD outbreak in particular sparked innovation, resulting in the first European- and US-approved EBOV vaccines [6]. Additionally, a randomized controlled phase II/III trial (“PALM”) conducted during this outbreak indicated improved clinical outcomes in certain patient cohorts receiving monoclonal antibody mAb114 or monoclonal antibody cocktail REGN-EB3 [7]. Remdesivir, a nucleoside analog that was highly efficacious in non-human primate models of EVD [8], had little effect on patient outcome [7]. Thus, EVD patient therapy has been limited to the use of antibodies, which do not penetrate immune-privileged sites (i.e., brain, eyes, placenta, and testes) known to harbor EBOV in some EVD survivors [9] and which may result in the evolution of EBOV escape mutants [10]. Small molecules may have logistical advantages over antibodies with regard to long-term storage, transport to remote locations, and patient administration and therefore could be alternatives to antibodies or could be part of combinatorial therapies with antibodies [11].

An ideal filoviral therapeutic is virus specific enough to minimize off-target effects but broad enough to target multiple aspects of a particular step of the virus life cycle, multiple steps of the virus life cycle, and/or several closely related viruses [12]. For instance, REGN-EB3 is a cocktail of three monoclonal antibodies, all of which affect EBOV cell entry by targeting distinct GP_1,2_ sites [13]. In the absence of ideal therapeutics, combinations of therapeutics of different classes that, ideally, work synergistically may be considered [14], although combinatorial products face more challenging regulatory hurdles than single products.

Most small molecules that have proven highly efficacious in non-human primate models of EVD and/or have been evaluated in clinical trials are nucleoside analogs (e.g., favipiravir [15,16,17] and remdesivir [7]). Typically, these molecules are associated with a single mechanism of action, i.e., the inhibition of virus replication and/or transcription through interference with the L-contained RdRp activity [8,18,19].

**Figure 1:**
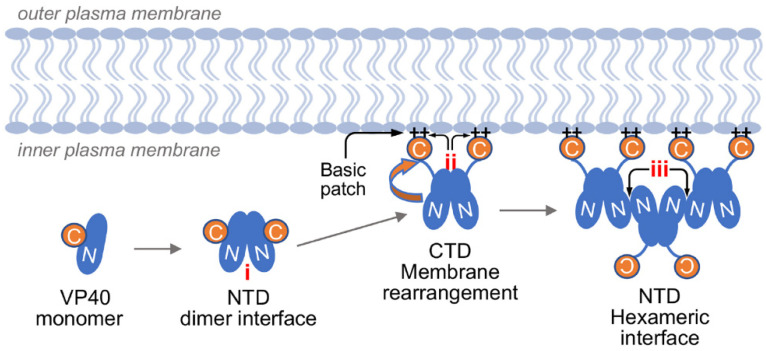
This cartoon schematic illustrates EBOV VP40–VP40 interaction phases crucial for viral particle formation: (i) VP40 NTD homodimers are formed. Once at the plasma membrane, VP40 rearrangement occurs through (ii) a basic patch containing six lysine residues. This rearrangement triggers exposure of (iii) an NTD surface that interacts at the hexameric interface between VP40 molecules necessary to form the viral matrix.

To narrow the gap in current EVD therapeutic availability, we sought to develop an assay to identify small molecules that target steps of the EBOV life cycle other than virion entry or replication/transcription. The last step in the EBOV replication cycle includes particle assembly, ribonucleoprotein (RNP) complex packaging, and virion release from the host cell membrane [1]. This process is mediated by EBOV matrix protein VP40, a multifunctional protein [20,21]. The VP40 monomer consists of distinct N-terminal domains (NTDs) and C-terminal domains (CTDs) that are joined through a flexible linker [22]. NTD–NTD interactions can lead to the formation of cyclic octamers that remain in the cytosol and bind RNA for regulation of EBOV replication and transcription [20,22,23,24,25,26,27,28,29,30,31,32,33,34,35]. VP40 requires three crucial interactions to promote formation of viral particles (Figure 1): VP40 must first form homodimers via NTD–NTD interactions for which residue L117 is crucial; such dimers accumulate at the plasma membrane due to VP40 domain rearrangement by which the CTD is flipped away from the NTD. This rearrangement exposes a basic patch comprised of six lysine residues (221, 224, 225, 270, 274, and 275) that interact with the inner membrane. This CTD juxtaposition exposes an additional NTD surface containing a conserved W95 that forms a secondary NTD–NTD interaction, which results in hexameric VP40 moieties. Interactions among VP40 hexamers form the viral matrix [20]. VP40 alone undergoes these interactions and thereby forms virion-like particles (VLPs) that are secreted from producer cells [20].

Our assay focused on VP40 because it is absolutely required for EBOV particle production [25]. Additionally, single and double mutants in the four discrete interactions are known to completely obstruct oligomerization and VLP formation [25]. Our hypothesis was that small-molecule disruption of this crucial nucleation for higher-order assembly of VP40 would inhibit virion formation [20,30]. Therefore, we designed a fluorescent live-cell assay based solely on EBOV VP40 expression to screen for compounds that could disrupt membrane formation of VP40-based VLPs; this disruption would directly affect the ability of VP40 to form VLPs on the cell surface and would be identified by the screening.

We identified a nucleoside analog, sangivamycin, that inhibited both EBOV VP40 association with the cell membrane and cellular virion-like particle release. As expected from these results, sangivamycin inhibited the replication of EBOV but, surprisingly, it also inhibited the replication of EBOV’s close relative, MARV, as well as the unrelated Lassa virus (LASV), cowpox virus (CPXV), and vaccinia virus (VACV), which do not express VP40 orthologs. Using an EBOV minigenome assay that does not encode or express VP40, we demonstrate that these broad-spectrum effects are likely due to a highly efficient secondary interaction with RdRps or other viral proteins required for viral replication and/or transcription. These data indicate that the VP40-based screening is suitable for the identification of novel EBOV MCMs and that sangivamycin could potentially be developed as a broad-spectrum antiviral once its mechanism of action is further clarified.

## 2. Materials and Methods

### 2.1. Cells and Cell Culture Conditions

Grivet (*Chlorocebus aethiops*) Vero E6 cells (NR596, BEI Resources, Manassas, VA, USA), human embryonic kidney (HEK) 293T cells (ATCC, Manassas, VA, USA) 293T/T17 cells (BEI Resources, Manassas, VA, USA), and human hepatocarcinoma (Huh7) cells (BEI Resources, Manassas, VA, USA) were maintained at 37 °C and 5% CO_2_ in Dulbecco’s Modified Eagle’s Medium (DMEM) (Life Technologies, Carlsbad, CA, USA) containing 10% heat-inactivated fetal bovine serum (FBS). Human monocyte-derived macrophages (MDMs) were differentiated from CD14^+^ monocytes and cultured as previously described [36,37].

### 2.2. Fluorescent VP40 Oligomerization Assay

For production of EBOV VP40, HEK 293T cells were plated at 10,000 cells per well in 384-well black-walled clear-bottom plates (BD Falcon, Corning, NY, USA). The next day, cells were transfected using TurboFect Transfection Reagent (Thermo Fisher Scientific, Waltham, MA, USA) with pIRES-P plasmids [38] encoding an EBOV/Yambuku-Mayinga VP40 fused to enhanced green fluorescent protein (eGFP) and V5 tag (eGFP-V5-VP40 wild type, henceforth referred to as VP40 WT) or a VP40 mutant unable to dimerize (eGFP-V5-VP40 L117R, henceforth referred to as VP40 L117R, and used in the high-throughput screening [HTS] as a positive control for non-oligomerized VP40). The simian virus 5 epitope (V5) tag peptide sequence (amino acids IPNPLLGLDST) and anti-V5 epitope mouse monoclonal antibody (Life Technologies) enabled western blot monitoring of the expression of full-length VP40 WT and L117R proteins and their presence in VLPs.

Four hours after transfection, cells were exposed to various concentrations of small molecules dissolved in dimethyl sulfoxide (DMSO) (Sigma-Aldrich, St. Louis, MO, USA) using a liquid handling robot (Janus Scientific Inc., Fairfield, CA, USA) and pin tools (PerkinElmer, Waltham, MA, USA). The National Cancer Institute (NCI) open repository compound library (2954 compounds) was evaluated in the primary screening at an initial concentration of 10 µM and follow-up quantitative (q) HTS in dose ranges from 30 µM to 1.2 nM. Cells were incubated with small molecules for 16 h, fixed in 4% paraformaldehyde (Electron Microscopy Sciences [EMS], Hatfield, PA, USA); nuclei were stained with Hoechst 33342 (Thermo Fisher Scientific), and cells were washed with phosphate-buffered saline (PBS) (Fisher Scientific, Hampton, NH, USA). Cell imaging was performed with a Synergy 4 plate reader (BioTek, Winooski, VT, USA). Inhibitors of VP40 oligomerization were initially identified as those small-molecule treatments resulting in enhanced green fluorescent protein (eGFP) fluorescence intensity reductions in the range of the diffuse fluorescence quantified for the positive control. Cells in wells associated with such reductions were further characterized by fluorescent imaging using an IX81 inverted microscope (Olympus Life Science, Waltham, MA, USA), a fluorescein isothiocyanate (FITC) filter cube, and an ORCA-05G digital CCD camera (Hamamatsu Photonics, Hamamatsu City, Shizuoka, Japan) to capture images of cellular eGFP and Hoechst 33342 signals at 10× magnification. A “hit” was defined as an inhibitor that reduced VP40 peri-membrane (“ring”) fluorescence commensurate with the appearance of homogeneous cytoplasmic fluorescent signal. The cytotoxicity of each hit was further assessed by visualization of cell nuclei via Hoechst 33342 staining. As a confirmatory measure, cell viability for each hit was measured using the CellTiter-Glo Luminescent Cell Viability Assay (Promega, Madison, WI, USA). A selectivity index (SI), defined as the ratio of the drug concentration required to reduce cell viability by 50% against the half maximal effective concentration (CC_50_:EC_50_), was calculated for each hit.

### 2.3. VLP Isolation and Western Blotting

HEK 293T cells transfected with TurboFect in six-well plates at 500,000 cells per well (BD Falcon, Corning, NY, USA) with 2 μg eGFP-V5-VP40 WT or eGFP-V5-APOBEC3G(A3G) were treated with compounds at multiple concentrations (0, 37.5, 75, 150, and 300 nM) in DMSO, as described in Section 2.2. DMSO-treated cells transfected with VP40 L117R positive control were included for comparison, final 0.25% DMSO. After 24 h, VP40 WT- and L117R-transfected cell culture media were harvested for analysis of secreted VLPs, and cell lysates were also collected in reporter lysis buffer (Promega, Madison, WI, USA). Culture media for each condition were filtered using a 0.45 µm filter to remove cell debris, and effluents were collected. VLPs were pelleted from the effluents by sedimentation through a 20% sucrose cushion by ultracentrifugation (100,000× *g* for 2 h). The pelleted VLPs were subjected to sodium dodecyl sulfate-polyacrylamide gel electrophoresis (SDS-PAGE), and western blotting with an anti-V5 antibody (Life Technologies) at a 1:4000 dilution was used to detect VP40 WT or VP40 L117R positive control. Additionally, lysates from treated cells were compared to untreated cell lysates by western blot to assess VP40 WT and VP40 L117R positive control abundance. Western blots were probed in parallel with anti-actin beta antibody (Sigma-Aldrich) at a 1:2000 dilution to confirm that similar amounts of each cell extract had been loaded and resolved by SDS-PAGE and western blot transferred to nitrocellulose membrane. Cells transfected with eGFP-V5-A3G were imaged, as described in Section 2.2.

### 2.4. Verification of Hits against Infectious Viruses

The infectious virus cell-based screening was performed as described previously [39]. Vero E6 cells were used to test small-molecule viral inhibitors of multiple and taxonomically distinct viruses. All viruses were grown at 37 °C and 5% CO_2_:

- *Bunyavirales*: *Arenaviridae*: Lassa virus strain Josiah (LASV). LASV (IRF0193; L segment, Genbank #KY425632.1; S segment, Genbank #KY425638.1) was grown in Vero E6 cells with alpha minimum essential media (MEM) (Thermo Fisher Scientific) containing 2% FBS (SAFC Biosciences, Lenexa, KS, USA) for 5 d. This working stock originated from a Centers for Disease Control and Prevention (CDC) isolate (#800789);
- *Chitovirales*: *Poxviridae*: recombinant cowpox virus expressing GFP (rCPXV-GFP) (seed stock received from United States Army Medical Research Institute of Infectious Diseases [USAMRIID]) [40] and recombinant vaccinia virus expressing GFP (rVACV-GFP) (seed stock received from Dr. Bernard Moss, National Institutes of Health [NIH] National Institute of Allergy and Infectious Diseases [NIAID]) [41]. Viruses were grown in Vero E6 cells with DMEM (Thermo Fisher Scientific) containing 2% FBS as previously described [42]; and
- *Mononegavirales*: *Filoviridae*: Ebola virus/H.sapiens-tc/GIN/2014/Makona-C05 (EBOV) and Marburg virus/H. sapiens-tc/AGO/2005/Angola-1379v (MARV). EBOV (IRF0165, Genbank #KY425645.1) was grown in Vero E6 cells with alpha MEM media containing 5% FBS for 7 d. The seed virus from which it originated was obtained from Public Health Agency of Canada (PHAC). MARV (IRF0202, Biosample #SAMN05916381) was grown in Vero E6 cells with alpha MEM containing 2% FBS for 5 d. The seed virus originated from University of Texas Medical Branch (UTMB).

Small molecules were also tested against EBOV, LASV, MARV, rCPXV-GFP, and rVACV-GFP in Huh7 cells. MDMs were used to further test the most promising hit, sangivamycin, against EBOV.

One day prior to assay performance, black opaque (for cytotoxicity) or clear-bottom 96-well (for efficacy) Operetta plates (Greiner Bio-One, Monroe, NC, USA) were seeded with Huh7 (30,000 cells per well), Vero E6 (30,000 cells per well), or MDMs (100,000 cells per well). Compounds were dissolved in DMSO to a concentration of 0.05% for stock solutions. For each assay, an 8-point dose-response curve with two-fold compound dilutions (600 to 4.69 nM) was prepared. Each dose was evaluated in triplicate. Toremifene citrate (T7204-25MG, Sigma-Aldrich) served as the positive control antiviral compound for EBOV, MARV, and LASV [14,43,44]; and cytosine β-D-arabinofuranoside (C1768-1G, Sigma-Aldrich) served as the positive control for both the rCPXV-GFP and rVACV-GFP assays [45,46]. Untreated cells were included as additional controls.

Cells were pre-treated with each dose of the compounds for 1 h prior to virus exposure. Exposures were performed at the following multiplicities of infection (MOIs): 0.1 or 0.5 MOI (EBOV), 0.5 MOI (LASV), 0.5 MOI (MARV), and 0.1 MOI (rCPXV-GFP and rVACV-GFP). For time-of-addition studies, virus was added first and compounds were added 1, 2, 4, 8, and 24 h after virus exposure.

After 24 h (rCPXV-GFP and rVACV-GFP) or 48 h (EBOV, LASV, and MARV), cells were fixed with 10% neutral-buffered formalin, washed with PBS, and blocked with PBS containing 2% *w*/*v* of bovine serum albumin (BSA) (Sigma-Aldrich).

Cells were then stained with primary antibodies corresponding to the virus used for infection:

- Anti-EBOV-VP40 (BMDO4B007 AE11, courtesy of USAMRIID, Fort Detrick, Frederick, MD, USA [47]) at a dilution of 1:4000;
- Anti-MARV-VP40 (0203-012, Integrated Biotherapeutics, Rockville, MD, USA) at a dilution of 1:4000; and
- Anti-LASV-GP (L-52-216-7, USAMRIID [48]) at a dilution of 1:3000.

Following PBS washes, cells were stained with peroxidase-labeled goat anti-mouse secondary antibody diluted at 1:4000 (5220-0339, SeraCare, Milford, MA, USA). Chemiluminescence was quantified using an Infinite M1000 microplate reader (Tecan, Morrisville, NC, USA). Fluorescence (for GFP-tagged viruses) was measured using an Operetta High-Content Imaging System (PerkinElmer, Waltham, MA, USA). Each experiment was run in duplicate (two plates) and repeated at least twice on separate days.

Sangivamycin was also tested against human immunodeficiency virus 1 (HIV-1) particles pseudotyped with vesicular stomatitis Indiana virus (VSIV) glycoprotein (G) instead of HIV-1 gp41/gp120 trimers. For pseudotype production, the proviral DNA plasmid pDHIV3-GFP was used, which contains all HIV-1 genes except *nef* (replaced with a gene encoding eGFP) and *env*. HEK 293T cells (producer cells) were transfected with the proviral vector and pVSV-G, a plasmid encoding VSIV G using FuGENE HD (Promega, Madison, WI, USA). Two μg total of proviral DNA:pVSV-G were added to cells at a ratio of 1:0.5, as described previously [49]. Pseudotype producer cells were dosed with sangivamycin in DMSO (0.25% final) 4 h after transfection, and HIV-1 pseudotypes were harvested 24 h later by filtering the media through a 0.45-micron syringe filter. Viral load was normalized with an HIV-1 p24 ELISA (PerkinElmer, Waltham, MA, USA). Equal loads of pseudotypes (500 pg p24) were added for 48 h to 10,000 cells per well TZM-bl reporter cells [50], which express firefly luciferase from the HIV-1-LTR promoter. Thus, pseudotype transduction efficiency at 48 h could be measured as relative light units (RLU) using a VICTOR^3^ Multilabel Plate Reader (PerkinElmer, Waltham, MA, USA) after cells were treated with Steady-Glo Reagent (Promega, Madison, WI, USA). Sangivamycin was added to both the producer cells and reporter cells, ranging from 600 to 37.5 nM in DMSO.

Compound cytotoxicity across the tested dose range was determined in parallel using treated but mock-exposed cells (drug and media only). The CellTiter-Glo Luminescent Cell Viability Assay (Promega) was conducted 48 h following compound treatment according to the manufacturer’s instructions. Briefly, at 48 h after drug treatment, 50 µL of the CellTiter-Glo reagent were added to each well of the plate. After 10 min of incubation at room temperature to allow maximum cell lysis, luminescence was measured on an Infinite M1000 microplate reader (Tecan). Cytotoxicity also was evaluated based on changes in chromatin domain nuclei staining (Hoechst 33342, Thermo Fisher Scientific) of compound-treated cells relative to that observed in untreated cells using the Operetta High-Content Imaging System. All plates were assessed for signal-to-noise ratios and Z’-factor scores for quality-control purposes.

### 2.5. Minigenome Assay

The standard EBOV minigenome system was obtained from Dr. Elke Mühlberger (Boston University School of Medicine, Boston, MA, USA). This system uses a reporter gene encoding firefly luciferase and works by cellular transfection of a minigenome reporter plasmid (3E5E-Luci) containing the reporter gene flanked by the EBOV 3′ trailer and 5′ leader as well as the NP 5′ and L 3′ untranslated region sequences [51], four plasmids expressing the EBOV RNP complex components (pCAGGS NP, VP35, VP30, and L) under a chicken actin beta promoter, and a sixth pCAGGS plasmid encoding T7 polymerase. Co-transfection of all six plasmids results in replication of the minigenome and, thereby, reporter gene expression in the absence of particle formation due to the absence of VP40 in the system [52].

T75 flasks (Corning, Corning, NY, USA) were seeded with 5 × 10^6^ HEK 293T/T17 cells per flask containing 12 mL of medium each. The next day, 56 µL of Lipofectamine 3000 Transfection Reagent (Thermo Fisher Scientific) was diluted in 1.5 mL of Opti-MEM Reduced-Serum Medium (Life Technologies). A mixture of the six EBOV minigenome plasmids (4.3 µg of pCAGGS-NP, 2.2 µg of pCAGGS-VP35, 1.7 µg of pCAGGS-VP30, 8.6 µg of pCAGGS-L, 3.4 µg/µL of 3E5E-Luci reporter plasmid, and 3.4 µg/µL of pCAGGS-T7) was prepared in Opti-MEM. The negative control plasmid mixture excluded the plasmid encoding VP35 and instead included an empty vector. Prepared plasmids and transfection reagent were mixed and incubated for 10 to 20 min at room temperature. From each T75 flask, 6 mL of media were removed, and plasmid transfection or negative control mixtures were added and incubated for 24 h. After incubation, cells were detached from the flask by enzyme-free Cell Dissociation Buffer (Thermo Fisher Scientific) and resuspended in DMEM without phenol red (Thermo Fisher Scientific). Cells were re-seeded in 96-well plates (Corning) at 30,000 cells per well in 50 μL of medium and allowed to settle for 1 h. Black and white opaque plates were used for cytotoxicity and luciferase measurements, respectively. Each plate was seeded with 11 columns of cells transfected with minigenome plasmid mixture and one column of cells with negative control plasmid mixture. After settling for 1 h, cells were treated with sangivamycin (300 to 9.4 nM) or remdesivir (3000 to 94 nM, Biosynth International, Inc., San Diego, CA, USA; catalog #AG170167) in a six-point, two-fold dilution scheme in triplicate on three separate plates. Cells were also treated with constant ratios of sangivamycin (300 to 4.7 nM) to remdesivir (6000 to 4.7 nM) at 1:2.5, 1:5, 1:10, 1:20, and 1:40 in triplicate for each concentration. The remaining wells on each plate were split between untreated negative control mixture-transfected cells and untreated cells transfected with a complete minigenome plasmid mixture. At 24 h, luciferase activity and cytotoxicity were measured (one plate per assay per time point). Steady-Glo Reagent (Promega) was used to develop luciferase plates for measurement according to manufacturer’s instructions. At 24 h post-treatment, firefly luciferase substrate was added, and the luciferase signals were measured using an Infinite M1000 Microplate Reader (Tecan). At 24 h after drug treatment, the CellTiter-Glo Luminescence Cell Viability Assay was used to measure cytotoxicity, as described in Section 2.4. Percentage cytotoxicity and inhibition was determined relative to the untreated controls in each plate.

## 3. Results

### 3.1. EBOV VP40 Membrane Localization Is Disrupted by Sangivamycin

To identify small molecules that could interrupt assembly/budding of EBOV, we screened live cells expressing eGFP-tagged EBOV VP40 (VP40 WT) in the presence of the molecules for changes in VP40 localization to the inner surface of the cell plasma membrane and eGFP fluorescence pattern changes compared to EBOV VP40 in the absence of small molecules (primary screening).

**Figure 2:**
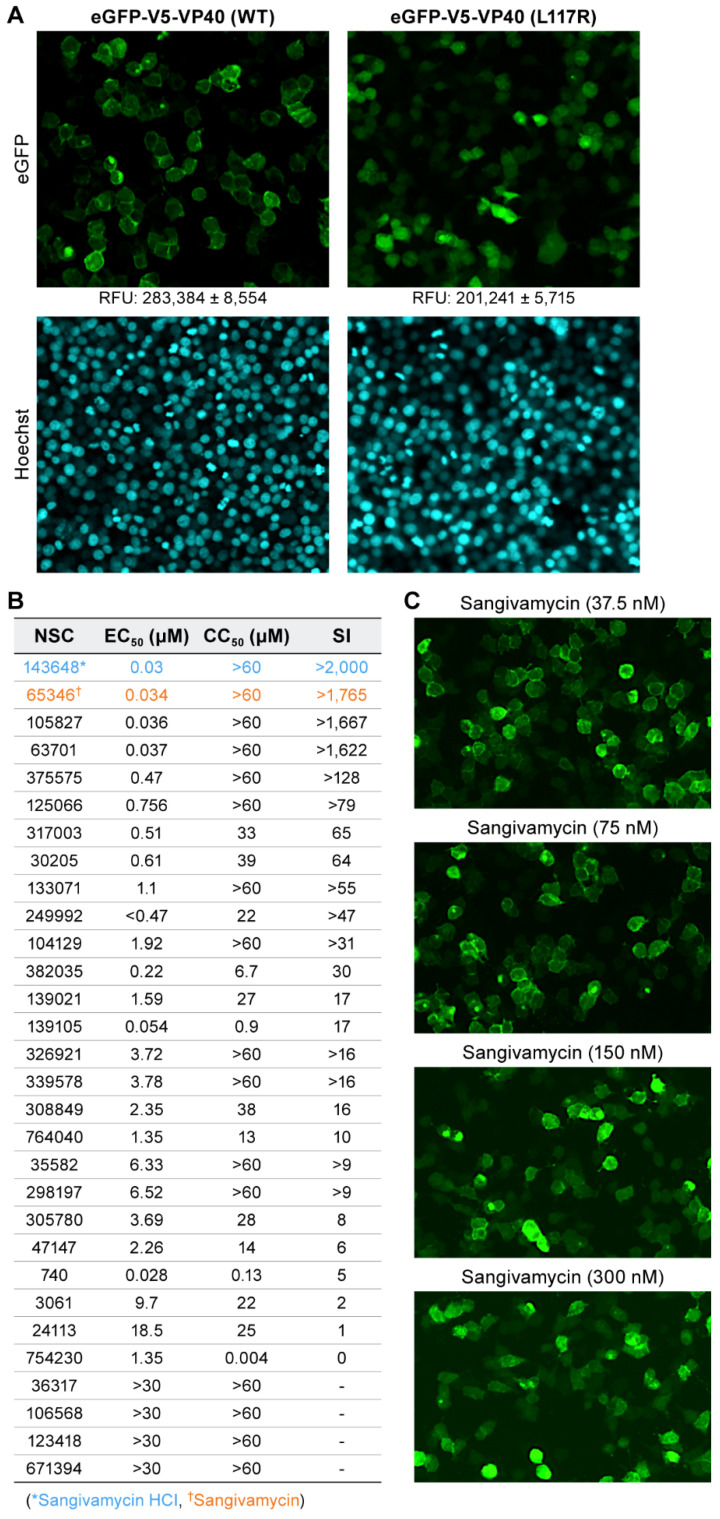
EBOV VP40 membrane localization is disrupted by sangivamycin. (**A**) Disruption of VP40 membrane association via the introduction of the L117R mutation (VP40 L117R positive control). Relative fluorescence units (RFUs) are shown below the eGFP images at 10× magnification. Lower RFU values for hits were then phenotypically confirmed via VP40 membrane association and fluorescent cellular rings for VP40 WT (top left panel). VP40 L117R positive control prevents initial VP40 dimerization and virion-like particle (VLP) formation leading to diffuse cellular fluorescence with lower RFU (top right panel). Hoechst 33342 DNA staining for chromatin in nuclei was used as an initial indicator of cell health (bottom panels). (**B**) The table lists the top 30 hits by their NCI assigned NSC numbers. Sangivamycin as either its HCl salt (NSC 143648) or free base (NSC 65346) impairs EBOV VP40 localization to the inner plasma membrane. Listed are the half maximal effective concentration (EC_50_), drug concentration required to reduce cell viability by 50% (CC_50_), and selectivity index (SI) values of the 30 hits identified in the primary screening and evaluated in the secondary screening. EC_50_ values were quantified by plate reads compared to VP40 WT and VP40 L117R positive control values. CC_50_ values were measured with CellTiter-Glo as relative luciferase units compared to VP40 WT and VP40 L117R positive control and SI was calculated by CC_50_/EC_50_. Although initial quantification was performed by plate reader, all hits were confirmed by a phenotypic shift from fluorescent rings to homogenous expression by cellular imaging. (**C**) Representative fluorescent images of VP40 WT treated with 37.5, 75, 150, and 300 nM sangivamycin at 10× magnification.

**Figure 3:**
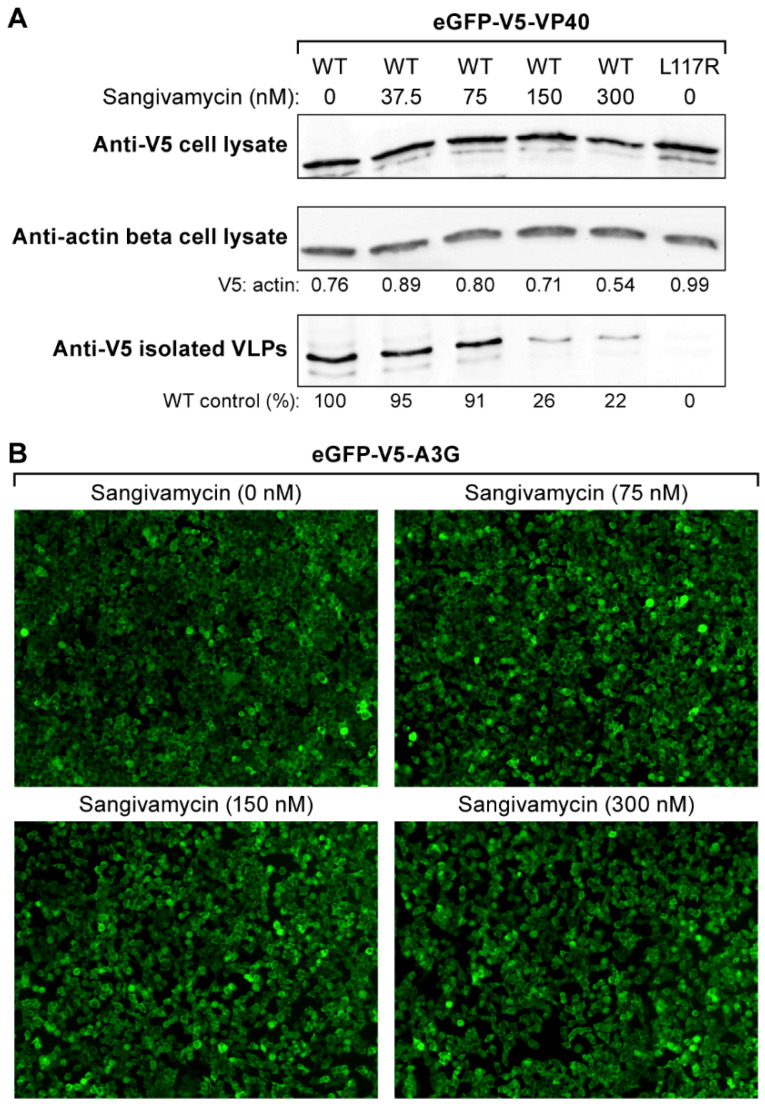
Sangivamycin decreases VLP release but does not alter VP40 abundance or cellular transcription/translation. (**A**) Shown are western blots performed on cell lysates and VLPs isolated from cell supernatants after transfection of plasmids encoding VP40 WT and VP40 L117R positive control. Sangivamycin was tested at 0, 37.5, 75, 150, and 300 nM. western blots show amounts of VP40 WT detected in cell lysates based on V5 epitope reactivity (VP40) (top) compared to anti-actin beta reactivity (V5: actin ratio shown) (middle). The bottom panel shows VLPs isolated from supernatant following sangivamycin treatment compared to VP40 WT control (bottom left lane). The VP40 L117R positive control did not result in VLP production (bottom right lane) despite slightly greater overall cellular abundance compared to VP40 WT (top right vs left lane). (**B**) Images showing the cytoplasmic protein A3G linked to eGFP expressed from the same plasmid as eGFP-V5-VP40 in the presence of sangivamycin at 0, 37.5, 75, 150, and 300 nM.

In the absence of small molecules, VP40 WT membrane localization can be visualized as a bright fluorescent lining of cell membranes (Figure 2A, top left panel). In contrast, an L117R point mutation in VP40 WT (positive control) that abolishes VP40 oligomerization by preventing initial VP40 NTD dimerization manifests as diffuse cytoplasmic fluorescence (Figure 2A, top right panel). Despite equivalent expression (as assessed by western blotting; see Figure 3), the diffuse cytoplasmic fluorescent signal from VP40 L117R positive control appeared less intense than the peri-inner membrane fluorescence of VP40 WT, with a Z’-factor of 0.5, calculated from RFU and standard deviation values in Figure 2A. This finding is consistent with a previous report on the effect of the L117R mutation on VP40 and confirmed the ability of our assay to discern ablation of VP40–VP40 interaction at the cell membrane [20]. Extrapolating from these results, we hypothesized that any small molecule efficient at disrupting wild-type VP40 self-interaction would result in altered fluorescence characteristics approaching those similar to that of VP40 L117R positive control.

In the primary screening of the NCI library of 2954 small molecules, 30 hits were identified. Next, these hits were screened by quantitative high-throughput screening (qHTS). Through qHTS, we selected hits with dose-dependent effects on VP40 membrane localization in a dose range of 30 µM to 1.2 nM. Thirteen of the initial hits were dose dependent in qHTS, with 50% effective concentration (EC_50_) values less than 4 µM and 50% cytotoxic concentration (CC_50_) values greater than 30 µM (Figure 2B). Hits were further triaged based on their SI, which was defined as the EC_50_:CC_50_ ratio. Four hits had SI values greater than 1600 (Figure 2B). Since these hits were structurally related, we selected the hit with the lowest EC_50_ and greatest SI (NSC 143648, sangivamycin HCl and NSC 65346, sangivamycin; see Figure 2B). In the qHTS, VP40 subcellular localization (shown as fluorescence expression at the cell membrane) clearly decreased and became more diffuse throughout the cytoplasm with increasing concentrations of sangivamycin. At 150 and 300 nM, sangivamycin images showed homogenous VP40 subcellular localization, indicating the ablation of VP40–VP40 membrane localization (Figure 2C).

### 3.2. Sangivamycin Decreases VLP Release but Does Not Alter VP40 Abundance or Cellular Transcription/Translation

We confirmed the effect of sangivamycin on VP40 membrane localization by quantifying the release of VLPs from the sangivamycin-treated cells (treated at concentrations of 37.5, 75, 150, and 300 nM) versus untreated cells. If VP40 cannot localize at the cellular membrane to form VLPs, VLPs should not be released in the cell supernatant. VLPs isolated from cell supernatants and cell lysates were collected for western blots 24 h after transfection with VP40 WT or VP40 L117R positive control plasmids. VLPs and cell lysates for each treatment group were subjected to SDS-PAGE and western blotted with anti-V5 (VP40) or anti-actin beta antibody. The blots were densitometrically scanned for semi-quantitative analysis of VLPs and cell lysates based on relative VP40 abundance.

Sangivamycin reduced the recovery of V5-tagged VP40 as secreted VLPs in a dose-dependent manner. The reduction in VLPs released from treated cells at and above 150 nM (Figure 3A, bottom) was consistent with the reduction in eGFP-V5-VP40 inner membrane localization observed at the same sangivamycin concentrations (Figure 2C). As expected, cell culture supernatants from cells transfected with plasmid encoding VP40 L117R positive control did not contain detectable V5-tagged VP40, indicating that VLPs had not been released (Figure 3A, bottom right).

To exclude the possibility that the reduction in VLP release was due to off-target inhibition of VP40 expression or alterations in VP40 protein turnover, we examined VP40 expression in cell lysates. The ratio of VP40 WT relative to cellular actin beta in cell lysates was similar among all treatment groups (Figure 3A, top and middle). The general lack of an effect of sangivamycin on transcription/translation was also confirmed by quantifying the abundance of an unrelated stably expressed eGFP linked protein in HEK 293T cells (Figure 3B).

### 3.3. Sangivamycin Inhibits the Replication of Multiple Viruses, Including EBOV and MARV

Having determined that sangivamycin is an effective antagonist of EBOV VP40 in viral particle assembly, we evaluated whether sangivamycin has antiviral activity against infectious EBOV in cell culture. Additionally, sangivamycin was tested in Huh7 and Vero E6 cells against multiple other viruses. Prior to virus exposure, cells were pre-treated for 1 h with sangivamycin. Pre-treatment involved the preparation of an eight-point dose-response curve with two-fold dilutions (600 to 4.7 nM). Cells were then exposed to EBOV, MARV, LASV, rCPXV-GFP, and rVACV-GFP. Plates were fixed after 24 or 48 h and developed via antibody staining specific for each virus (except for the recombinant viruses). Chemiluminescence of WT viruses was measured by staining with a peroxidase-labeled secondary antibody. GFP fluorescence was measured from the recombinant viruses. Cytotoxicity was measured with CellTiter-Glo assay that measures ATP levels to determine cell viability according to the manufacturer’s instructions.

**Figure 4:**
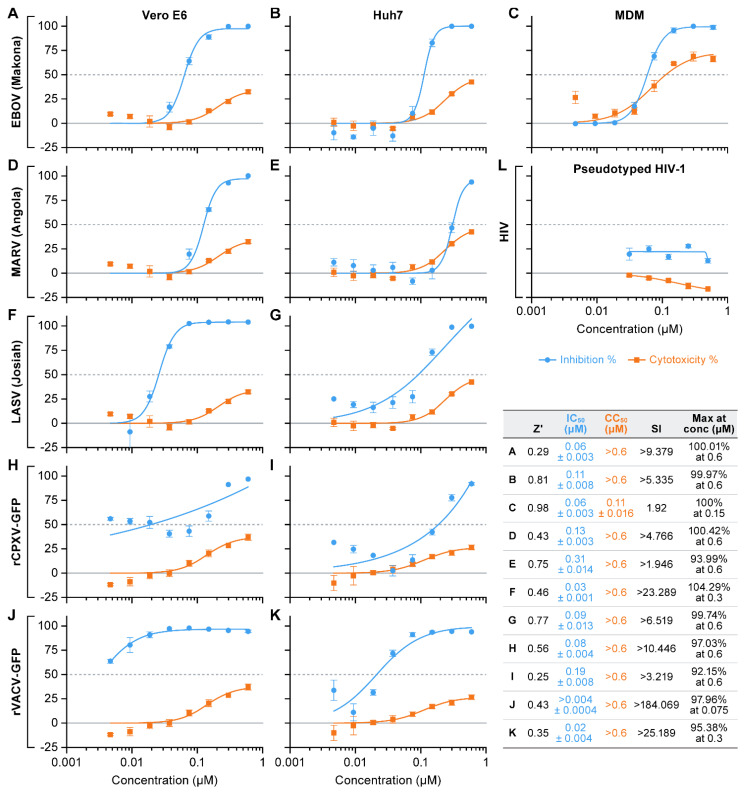
Sangivamycin inhibits the replication of multiple viruses, including EBOV and MARV. (**A**–**K**) Infectious virus cell-based enzyme-linked immunosorbent assays were performed to determine efficacy and cytotoxicity of sangivamycin in the presence of various viruses. Results are reported as percent inhibition and cytotoxicity relative to untreated controls. Results for each virus tested are shown. (**L**) HIV-1 pseudotype control, TZM-bl cells. Z’ values 0.2 to 1 were considered acceptable. Each concentration was run in triplicate. Plates were run on two separate occasions to ensure reproducibility and agreement between runs. Graphs shown above were generated from one of two runs.

Consistent with the data obtained from our primary screening, sangivamycin was effective against EBOV in Vero E6 and Huh7 cells, but surprisingly also was effective against MARV, rCPXV-GFP, and rVACV-GFP in both Vero E6 and Huh7 (Figure 4A,B,D–J). The sangivamycin IC_50_ values ranged from 0.1 to 0.3 µM for MARV to approximately 0.04 to 0.1 µM for EBOV. The compound did not affect HIV-1 pseudotype transduction in a 293T producer cells and a TZM-bl target cell-based system (Figure 4L).

Since sangivamycin was originally evaluated as an anti-cancer drug and since Huh7 and Vero E6 are immortalized cell lines, we evaluated whether sangivamycin would still be efficacious in a primary cell line [53]. Sangivamycin was active in MDMs against EBOV; however, higher cytotoxicity was also observed (Figure 4C), though the effect is likely not cytotoxicity but rather a cytostatic effect as reported in the literature [54,55,56], given that MDM cells remained on the plate albeit in lower abundance, presumably due to a lack of cell divisions (data not shown).

### 3.4. Sangivamycin Inhibits EBOV RNA Transcription/Replication

The broad antiviral effect of sangivamycin against viruses lacking a VP40 homolog or ortholog (rCPXV-GFP, rVACV-GFP, LASV) and the absence of acute cellular toxicity suggested that sangivamycin may interact with EBOV at sites other than VP40. We therefore evaluated sangivamycin efficacy in the absence of EBOV VP40 WT expression using an EBOV minigenome assay, which offers a measure of EBOV L (RdRp) activity. Cells were transfected simultaneously with plasmids expressing the EBOV RNP complex components NP, VP35, VP30, and L along with a plasmid expressing T7 polymerase and another plasmid containing the EBOV genomic 3′ and 5′ leaders flanking a luciferase reporter gene. Transcription of the reporter is driven by the RNP complex (after initial transcription from transfected plasmids by T7 polymerase) [52].

**Figure 5:**
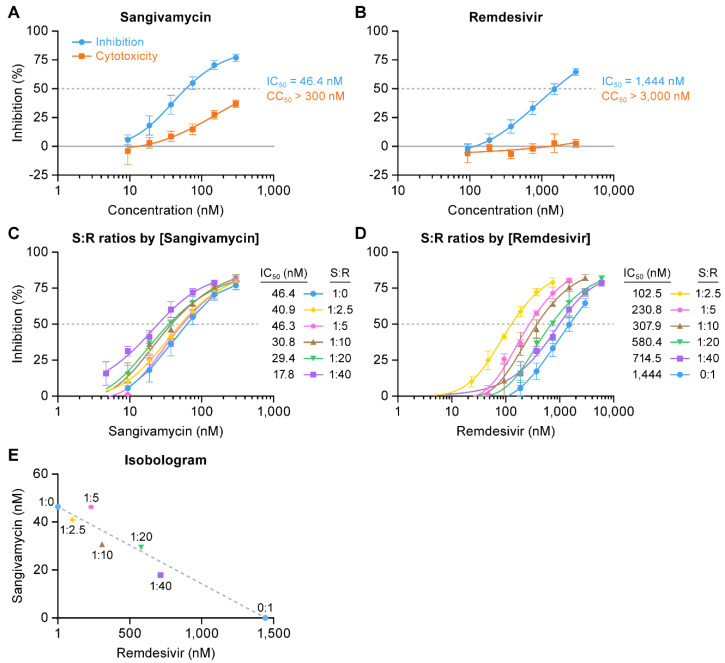
Sangivamycin inhibits EBOV RNA transcription/replication. Briefly, EBOV minigenome plasmids were first transfected in HEK 293T/17 cells. Cells were initially treated with (**A**) sangivamycin (300 to 9.4 nM) and (**B**) remdesivir (3000 to 94 nM) and cytotoxicity and luciferase activity were measured at 24 h. Based on the calculated IC_50_ values from the single drug tests in **A** and **B**, constant ratios of sangivamycin to remdesivir (S:R = 1:2.5, 1:5, 1:10, 1:20, 1:40) were set up for drug combination studies (**C**–**E**). The constant ratios were plotted relative to (**C**) sangivamycin concentration (1:0 represents sangivamycin alone from **A**) and (**D**) remdesivir concentration (0:1 represents remdesivir alone from **B**). (**E**) The isobologram shows the IC_50_ values calculated from the curves in **C** and **D** plotted on the y axis (values from **C**) and x axis (values from **D**) to assess the constant ratios relative to the additive line (dotted line) drawn between the IC_50_ values for sangivamycin and remdesivir alone calculated from curves **A** and **B**, respectively. All of the constant ratios are along the additive line within the 95% confidence interval for each point. Data for percent inhibition and cytotoxicity were determined based on a comparison to untreated negative control cells. Data represent means ± standard deviations (SD) of results from triplicate samples for the constant ratios and an n of 9 for sangivamycin- and remdesivir-alone treatments.

Treatment with sangivamycin yielded a measurable dose-dependent reduction in luciferase expression. Luciferase activity (expression) reduced greater than 80% of the activity quantified in untreated control cells at 24 h with an IC_50_ of 46.4 nM (Figure 5A). At the highest dose and 24 h post-exposure, dose-dependent cytotoxicity of sangivamycin was approximately 30%. Minigenome activity was also tested with remdesivir, which specifically targets EBOV L activity [57]. As expected, remdesivir was active in the minigenome assay, albeit with an IC_50_ of 1444 nM (Figure 5B). Because both remdesivir and sangivamycin are nucleoside analogs, we tested them in combination at several constant ratios relative to their respective IC_50_ values to determine whether they are synergistic, additive, or antagonistic against the EBOV L target. Increasing amounts of remdesivir relative to sangivamycin (Figure 5C) or increasing amounts of sangivamycin relative to remdesivir (Figure 5D) both lowered the IC_50_ of the respective treatments. These IC_50_ values were plotted for each constant ratio of sangivamycin to remdesivir (S:R) in an isobologram [58]. A straight line between the IC_50_ for sangivamycin alone (46.4 nM) on the y axis to the IC_50_ for remdesivir alone (1444 nM) on the x axis determined the additive line (Figure 5E, dotted line). IC_50_ values for the constant ratios of S:R were plotted along the additive line (Figure 5E). The results suggest that both compounds are acting on the same target and not affecting the activity of each other. IC_50_ values for the constant ratios significantly above the additive line would indicate that the compounds were antagonistic to each other in targeting EBOV L activity. On the other hand, if they were acting on different targets to affect the minigenome assay (e.g., a cellular target that indirectly affects the minigenome readout), then the combined treatment would have resulted in data that plotted below the additive line indicative of synergy.

### 3.5. Sangivamycin Inhibits EBOV up to 24 H after Addition Relative to Virus Exposure

**Figure 6:**
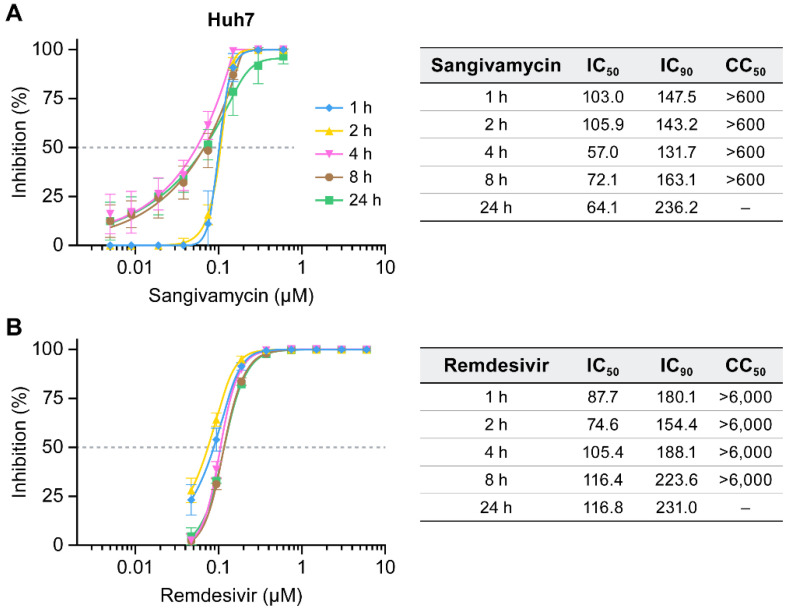
Sangivamycin inhibits EBOV up to 24 h after addition relative to virus exposure. Huh7 cells were infected with EBOV and sangivamycin (**A**) or remdesivir (**B**) were added to the cells at 1, 2, 4, 8 and 24 h after virus exposure. Results are reported as percent inhibition and cytotoxicity relative to untreated controls. Each concentration was run in triplicate.

To further show that sangivamycin is directly targeting specific EBOV processes, antiviral activity was measured in a time-of-addition experiment of sangivamycin relative to virus exposure of Huh7 cells. Both viral replication/transcription and VP40-dependent viral assembly processes occur late in the viral life cycle; therefore, it was expected that the antiviral activity would be unchanged up to 24 h after adding the drug relative to viral infection of the cells. On the other hand, if sangivamycin only affects viral entry or an indirect cellular process (and not late-stage viral processes directly), it is anticipated that diminishing effects on EBOV infectivity would occur at later time points of drug addition. At all time points of addition, sangivamycin’s IC_50_ values were similar or lower than those at later time points (Figure 6A). Remdesivir’s IC_50_ value changes were also less than significant (Figure 5B). This result was not surprising as remdesivir directly affects EBOV L-dependent replication and transcription late in the viral life cycle [57]. Notably, time-of-addition curves for remdesivir are overlaying, but the late addition of sangivamycin appears to lower the IC_50_.

## 4. Discussion

We developed a new assay to identify compounds that disrupt EBOV VP40 recruitment for viral particle assembly. VP40 is an abundant filovirus protein that is critical to the virus life cycle. The primary high-content, high-throughput assay was developed in cells expressing only fluorescent VP40 to identify compounds that prevented VP40 recruitment to the inner surface of the cell plasma membrane for viral particle assembly. The advantage of our cell-based assay is that initial hits are cell permeable, react with VP40 when expressed alone in a cellular context, and can easily be identified as overtly cytotoxic or not. We screened approximately 3000 compounds from the NCI open repository compound library and phenotypically confirmed hits that prevented VP40 membrane localization using cellular imaging. We first selected hits that caused dose-dependent inhibition of VP40 membrane localization. Hits were then vetted for low cytotoxicity, ability to inhibit VLP release, and antiviral activity against infectious virus in culture. Finally, hits were counter-screened using a minigenome assay, an assay excluding VP40, to determine whether hits targeted additional EBOV proteins and a time-of-addition test of viral infectivity to determine the stage of the viral life cycle that is affected.

**Figure 7:**
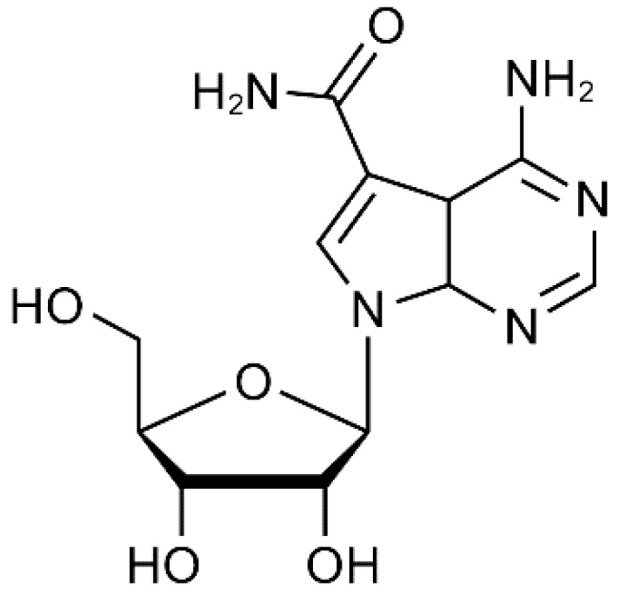
*Sangivamycin*: 4-amino-7-(β-D-ribofuranosyl)pyrrolo[2,3-*d*]pyrimidine-5-carboxamide (C_12_H_15_N_5_O_5_); Chemical Abstract Service #18417-89-5; molecular weight: 309.27.

Our results demonstrate that sangivamycin, an antibiotic derived from *Streptomyces* sp. and previously tested as an anti-cancer drug [59,60,61,62], is a potent inhibitor of a broad spectrum of viruses, thereby validating the VP40-based screening method. Sangivamycin’s broad-spectrum activity is perhaps unsurprising given that sangivamycin is an adenosine nucleoside analog [59] (Figure 7).

Remdesivir also is a nucleoside analog [8], but neither remdesivir nor GS-441524 (the parent nucleoside to remdesivir) affected VP40 perimembrane. Sangivamycin therefore is a dual EBOV antagonist in vitro, which may account for its particularly potent antiviral activity against EBOV. It is intriguing to consider that the reduction in IC_50_ upon late addition of sangivamycin in the time-of-addition assay (Figure 6A) is due to its dual targeting on viral replication and viral particle assembly and release whereas remdesivir has a single mode of action and its curves overlapped independent of time-of-addition (Figure 6B). Worth exploring in the future are whether sangivamycin is a dual antagonist of other ebolaviruses (in particular, Bundibugyo virus [BDBV] and SUDV) or MARV as well as the nature of the viral replication and/or transcription inhibition in the minigenome assay. L is the most likely target of the nucleoside analog since sangivamycin was additive with remdesivir when combined, but the AMP binding pocket in viral nucleoproteins is another possible target, as shown for another small molecule (PJ34) targeting coronaviruses [63]. Sangivamycin inhibits the replication of some viruses not tested here, such as herpes simplex viruses 1 and 2 (IC_50_ = 226 nM) [64,65,66], rhinoviruses (IC_50_ = 291–485 nM) [67], vesicular stomatitis Indiana virus and human parainfluenza virus 3 (IC_50_ = 65 nM), coxsackie virus B4 (IC_50_ = 129 nM), poliovirus 1 (IC_50_ = 226 nM), Sindbis virus (IC_50_ = 646 nM), vaccinia virus (IC_50_ = 65 nM), and reovirus type 1 (IC_50_ = 323 nM) [66]. These findings are consistent with the broad-spectrum nature of sangivamycin we identified against LASV, rVACV-GFP, and rCPXV-GFP.

Importantly, the antiviral concentrations required for filovirus inhibition did not seem to impact cellular RNA polymerase or protein expression in general. Although sangivamycin was not an effective clinical treatment for cancer when tested in the 1960s, its effectiveness against cancer cell line growth in vitro has led to multiple hypotheses for its mechanism of action in cancer cells. Since the clinical trials in the 1960s, many laboratories have studied the drug’s effect on translation machinery, cellular RNA and DNA polymerase function, and cellular kinases [53,59,60,68,69]. Sangivamycin has activity against all these cellular functions, but at substantially higher doses (micromolar) [53,59,68,69] than were determined to be effective against EBOV and other viruses in our study (nanomolar) and those previously published [64,65,66,67].

The most studied inhibitory mechanism of action for sangivamycin is against cellular kinases. Sangivamycin may inhibit cellular kinases that are overexpressed in certain cancer cells, most notably protein kinase C (PKC) and haspin, which may have an apoptotic effect in certain cancer cell types [e.g., pancreatic cancer cells, breast cancer MCF-AR cells, and primary effusion lymphoma (PEL) cells] [54,55,56]. On the other hand, sangivamycin is well tolerated in other cancer cells or normal cell types (e.g., normal pancreatic cells, Ramos cells, Burkitt lymphoma DG75 cells, and breast cancer MCF-WT cells), with cytostatic effects only becoming apparent at the high nanomolar to low micromolar dose range [54,55,56]. Although cell growth is affected at these higher concentrations, we observed that both MDMs and 293T cells remain attached and do not undergo apoptosis (data not shown). Numerous kinases are involved in EBOV replication [70,71,72,73,74,75,76]. It therefore must be acknowledged that some of them may be inhibited by sangivamycin, even at low concentrations, thereby influencing VP40 transport to the plasma membrane, VP40 oligomerization, or VP40 membrane association (VP40-specific), or may affect other parts of the EBOV life cycle (dual specificity, possibly targeting proteins other than L/RdRp).

In considering the in vitro data, we must also consider that sangivamycin was already tested in clinical trials in the 1960s for anti-cancer activity. The compound proved inactive against cancer but was well tolerated with daily, thrice-weekly, or weekly dosing in 40 patients (0.1 to 2.83 mg/kg total dose) [60]. Preclinical work archived at NCI revealed that sangivamycin was tested in African green monkeys and was tolerated for 10 d at 1.6 mg/kg/d (total dose 16 mg/kg) and 28 d at 0.4 mg/kg/d (total dose 11.2 mg/kg) [77]. To move forward as a filoviral therapeutic candidate, animal studies, including more in-depth pharmacokinetic and tolerability studies, should be performed to ensure that the effective concentration of sangivamycin can safely be administered.

Notably, in laboratory mice, sangivamycin appears to be retained in tissues for days following a single dose [78]. This retention suggests that sangivamycin could be a candidate for single or limited dosing. Assuming that slow metabolic turnover of sangivamycin occurs in humans, we have estimated the amount of sangivamycin in a two-compartment model for a 70-kg adult male (extracellular and cellular water volumes will be 42 L). Based on this estimate we calculated that doses already demonstrated to be safe in humans could be greater than 10-fold above the concentration needed to achieve an antiviral IC_90_.

In summary, we have described a novel EBOV VP40-based drug screening assay and demonstrated its suitability for MCM identification through the discovery of sangivamycin as a first in class of compounds that inhibits EBOV VP40 participation in virion assembly. The validation of the VP40 primary screening and critical path to identify hits will enable screening of larger small-molecule libraries to identify additional novel EBOV VP40 inhibitors.

## 5. Patents

Patents have been filed on Sangivamycin in the USA (PCT/US2017/060207), in Canada (06384.005CA1) and in Europe (WO2018089306) for Methods of Treating and Inhibiting Ebola Virus Infection.
